# Farmers' Perceptions of Preventing Antibiotic Resistance on Sheep and Beef Farms: Risk, Responsibility, and Action

**DOI:** 10.3389/fvets.2020.00524

**Published:** 2020-08-13

**Authors:** Charlotte Doidge, Annmarie Ruston, Fiona Lovatt, Chris Hudson, Lis King, Jasmeet Kaler

**Affiliations:** ^1^School of Veterinary Medicine and Science, University of Nottingham, Sutton Bonington, United Kingdom; ^2^Centre for Health and Social Care Research, Canterbury Christ Church University, Canterbury, United Kingdom; ^3^Agriculture and Horticulture Development Board, Stoneleigh Park, Kenilworth, United Kingdom

**Keywords:** antibiotic resistance, sheep, perceptions, behavior, risk, antibiotic use, cattle, antimicrobial resistance

## Abstract

Antibiotic resistance is one of the most serious public health risks facing humanity. The overuse of antibiotics in the treatment of infectious disease have been identified as sources of the global threat of antibiotic resistance. This paper examines how farmers perceive and manage risks associated with overuse of antibiotics. Specifically, the paper examines the role of habitus and risk in determining farmers' decisions to adopt national antibiotic reduction targets set by members of the Responsible Use of Medicines in Agriculture Alliance's Targets Task Force. Semi-structured interviews were conducted with 34 sheep and beef farmers in England and Wales. Farmers presented four scripts which illuminated reasons for limited adoption of the targets. The scripts presented the farmers as “good farmers” facing an emerging threat to their ontological security. Scripts suggested that they engaged in preventative measures but deflected responsibility for reducing antibiotic resistance to veterinarians and poorly run farms. This research provides valuable insights for policy makers and highlight the benefits of including social science research to support effective implementation.

## Introduction

Antibiotic resistance has been identified as one of the most serious public health risks facing humanity (1–3). It has been estimated that by 2050 it could result in 10 million deaths a year worldwide (1, 4). Other consequences of antibiotic resistance include increased duration of hospital stays, higher medical costs, and less sustainable food production resulting in food shortages (1). Risks posed by the overuse of antibiotics in the treatment of infectious disease, in both humans and animals (companion and production), have been identified as sources of this global threat (5, 6).

Currently in the United Kingdom (UK), overall veterinary antibiotic use estimates are derived from pharmaceutical sales data (7). Antibiotic sales are calculated by using a Population Correction Unit (PCU) as the denominator, which is the standard weight of animals at treatment time multiplied by total number of animals in the population. The tonnage of antibiotics sold is then divided by the PCU to get mg/PCU. The data is published annually by the Veterinary Medical Directorate (VMD) and the most recent publication reports that there was a reduction in the sales of veterinary antibiotics in recent years, from 62.5 mg/PCU in 2014, to 29.5 mg/PCU in 2018 (7). This was due to a large reduction in antibiotic sales indicated for use in pigs and poultry. The VMD report also includes sector specific antibiotic use for the salmon, trout, poultry meat, laying hen, and pig sectors using data submitted by farmers or veterinarians which is then collated by industry (7). Unlike the other sectors, the figures reported for the sheep and beef sectors uses small subset samples of prescription data to provide an indication of antibiotic use.

Sheep and beef production are typically extensive industries. There are over 72,000 breeding ewe holdings (8), and 59,000 beef cow holdings in the UK (9). The sheep sector is the largest livestock sector in the UK and accounts for 40% of the overall livestock biomass (10). The sheep and beef industries are highly interlinked, and the two livestock species often co-exist on farm holdings. This makes it particularly difficult to decipher use between the species when drugs licensed for use in both species are used. Furthermore, there are significantly fewer antibiotics that are specifically licensed for use in sheep, suggesting that there may be a need for the use of products not specifically licensed for sheep to be prescribed “under the cascade.” The cascade allows veterinarians to prescribe antibiotics that are not licensed for use in sheep providing that they can demonstrate that this is the most appropriate antibiotic for the situation. As of yet, there are no accurate estimates of data to determine the current antibiotic consumption by the sheep and beef sectors, and unlike the other sectors, at the time of writing, plans to centrally collate on-farm antibiotic usage data are still in development, and an ongoing focus of the Responsible Use of Medicines in Agriculture (RUMA) Targets Task Force and the Agriculture and Horticulture Development Board, which is funding the development (11). To support decisions on the most effective risk reduction strategies there have been calls for greater consistency, standardization, granularity and validation of surveillance data collected on antibiotic usage and antibiotic resistance (12).

UK policy responses have focused on the setting of national targets to reduce overall use of antibiotics and on implementing restrictions on some antibiotic drugs (4, 13, 14). The use of targets to reduce antibiotic resistance is based on the precautionary principle. The precautionary principle offers a solution to the problem of coping with the mixture of limited knowledge and ethical doubts with respect to the uncertain impact of technological developments, particularly in the fields of the environment and health. As such it provides a systematic way of coping with the irreducible uncertainties of decision making and thereby providing legitimation for policy (15). In the framing of public policy governments make risk choices, for example, the UK policy response to a range of food and environmental risks such as BSE, and *Escherichia coli* outbreaks has been risk averse with risk framed as contingent and uncertain (16). It has been suggested that the consequence of this approach, within the context of antibiotic resistance in agriculture, is that governments avoid risk by shifting responsibility to the farmers and veterinarians who become accountable for risk assessment and management (16).

The UK Government has published policies which have sought to set targets for the reduction in use of antibiotics in animal production (7, 17). This drive to ensure responsible use of antibiotics in agriculture has been led by RUMA. RUMA is an independent alliance made up of many organizations representing different livestock sectors and stages of the food chain. The group was set up in 1997 with the aim of promoting best practice in animal medicine use. In response to the increasing concerns around antibiotic resistance, they identified areas where antibiotic use could be reduced, refined, and replaced without compromising animal health and welfare (18). The RUMA guidelines state that all farmers have a responsibility for the health and welfare of the animals under their control and that they must take joint responsibility, with their veterinarians, in the discharge of correct and appropriate antibiotic treatment and care (18). They are accompanied by a set of industry-developed species-specific antibiotic usage targets for eight key UK livestock sectors. In particular, the sheep and beef sectors have identified a number of challenges to achieving target reductions including: no central or uniform system to collect data; low veterinary involvement on farms; difficulty collecting on farm data; separating usage between species and possible complacency within the sector, as many farms are extensive with a low numerical usage. Particular “hotspot” areas identified by the sheep sector as potential for high use of antibiotics were in the control of lameness, abortion, and neonatal losses. In beef cattle respiratory disease, calf scour, navel ill, mycoplasma, lameness, and calving related problems were identified as areas of potential high use (11). As a result, RUMA provided sector wide targets for the sheep and beef industry which included the target to reduce antibiotic use levels by 10% between 2016 and 2020 (18). In order for targets to be met, changes in behavior of both farmers and veterinarians is required.

### Risk Concepts in Social Science

Definitions of risk often specify it in terms of outcomes and probabilities that an adverse event(s) will occur within a stated time frame or as a result of a certain action. Risk assessment is the process of estimating both the probability that an event will occur and the probable magnitude of its adverse effects—economic, health, and safety or ecological over a specified period of time (19). Zinn (20) suggests that risk is used in two connected ways. Firstly, it is understood as a material or symbolic danger or harm or an alleged negative future event. Risk theorizing in this context concerns ways in which such dangers or harms are managed, prevented or attributed (or not) to decisions. Secondly, risk represents a specific form of managing uncertainty. The concept of risk is understood not as a harm or danger in governmentality but as a specific way to manage threats with calculative technologies (20). Additionally, risk refers to the possible occurrence of an adverse event, which can in turn be mathematically formalized as an expected loss. This approach to risk which is grounded in cognitive rationality involves collecting and analyzing knowledge/evidence and using it as part of a formal decision-making process to control uncertainties (21).

The way in which expert knowledge characterizes risk suggests that danger can be defined and managed or governed (22). Risk management then involves the prediction, analysis, and containment of risks so that overtime risks are converted to certainties (23). Governmentality focuses on ways in which risk should individually and collectively be managed (24). Lupton (24) argues that expert knowledges are pivotal to governmentality as they provide guidance and advice by which populations are surveyed, compared against norms and trained to conform with these norms. However, Wynne (25) argues that expert knowledge systems embody assumptions and modes of framing, using objectivist language, which implicitly treat the non-expert world as epistemically vacuous. He argues that they are incipient social prescriptions or vehicles of tacit social order. Thus, in this context, lay reflexivity is seen as having little instrumental value beyond the subjective and emotional world of its carriers when measured against a scientific world view (25). However, the rational, non-rational risk dichotomy has been criticized for not recognizing the limits of rational decision making, on the one hand and the knowledge and skills applied by lay people when making risk decisions, on the other hand (26, 27). Thus, assumptions about risk calculation through the cool deliberations of rational actors, abstracted from social and cultural settings and influences or from the impact of emotions have been called into question (23). Contexts in which judgements are made influence risky choices by both lay people and experts. Even where risk is described as “evidence based,” such as in medicine or the veterinary field, the type of objectivist calculations used and criteria that are selected for examination are necessarily confounded with moral judgements and real risks are transferred into cultural or symbolic risks (20).

Thus, at a micro level, knowledge production will be understood as situated in specific and contradictory contexts of the everyday. Such embodied knowledge includes pre-rational, aesthetic, emotional, and intuitional aspects of knowledge (20, 28). Social theories of risk emphasize the context within which decisions are made and locate individual risk decision making within social reality (16). They link together accounts of the origin, probability, and severity of the risk with views about feasible solutions (29). Risk perception is influenced by the characteristics of the risks, as well as by socio structural factors—people interpret their world through their mental models—and the knowledge systems of lay people offers a valid interpretation of risk (20). Perrow (30) suggests that cultures are dependent variables—that is, they are the results of other forces that develop to explain and legitimize practices and to provide ways of seeing and thinking that are compatible with current existence and experience. Lupton (26) suggests that judgements about what phenomena should be described as risky are influenced by social and cultural contexts, personal experience, and embodied sensations or emotions. She argues that emotion and risk are intersubjective and interpreted through a social and cultural lens and influenced by past experience and by the spaces and places we encounter every day. Lupton (26) uses the concept of the *emotion-risk assemblage* to acknowledge that emotions and risk judgements, rather than being located in the individual, are fluid, shared, and collective.

### Risk as Part of Farmers Habitus

The way in which Bourdieu sought to unpack the relationship between individual, agency, and wider social structures as determinants of individual behaviors or practice has been explored in the context of both farmer behaviors and human health (31–34). Crawshaw and Bunton (32) have argued that for Bourdieu actions of individuals and social groups incorporate influences from culture, traditions, and objective structures within society. These determine “practice” in unconscious and implicit ways and in turn normalize certain responses to present “a theory of practice” or habitus (32, 33). Individuals' own situated risk discourse are a product of habitus and can be characterized as practice with its own cultural logic. Shortall et al. (34) links Bourdieu's concepts of cultural capital and “habitus” to the concept of “good farmers.” Farmers' habitus involves striving to be good farmers incorporating their cultural capital—which includes prestigious skills, knowledge, and experiences into their everyday practice. Good farming can be exemplified through sound stockmanship, having the skills to assess animal well-being and/or disease status and by assessing and managing risk to animal health (34).

This study builds on the relationship between risk and habitus to gain an understanding of sheep and beef farmers' decisions and actions relating to reducing antibiotic usage, in order to support successful behavior change, policy implementation, and the reduction in the risk of antibiotic resistance.

## Methodology

This paper draws on a qualitative study developed as part of a larger longitudinal study which aimed to understand farmers' perceptions and use of antibiotics on sheep and beef farms in England and Wales. Semi-structured interviews were conducted with farmers on 34 farms of which eight were beef-only, four were sheep-only, and 22 were mixed species farms (Table 1).

**Table 1:** Participants' gender, farm location, and cattle and sheep numbers.

**Farm**	**Number interviewed on each farm**	**Gender**	**Farm location**	**Animal types and herd or flock size^a^**
1	2	2M	West Midlands	Medium beef herd
2	1	M	West Midlands	Medium beef herd, small sheep flock
3	1	M	West Midlands	Large beef herd, large sheep flock
4	1	M	Wales	Small beef herd, medium sheep flock
5	1	M	South West England	Large beef herd
6	1	M	West Midlands	Medium beef herd, small sheep flock
7	2	1M 1F	West Midlands	Large beef herd
8	1	M	South West England	Large beef herd
9	2	2M	South West England	Small beef herd, large sheep flock
10	1	M	Wales	Small beef herd, large sheep flock
11	1	M	Wales	Small beef herd, medium sheep flock
12	2	2M	Wales	Small sheep flock
13	1	M	Wales	Small beef herd, medium sheep flock
14	1	F	South East England	Medium beef herd, large sheep flock
15	1	F	Wales	Small beef herd, medium sheep flock
16	1	M	Wales	Small beef herd, medium sheep flock
17	1	M	Wales	Small beef herd, large sheep flock
18	1	M	Wales	Large beef herd, large sheep flock
19	1	M	West Midlands	Medium beef herd
20	2	1M 1F	Wales	Small beef herd, mediums sheep flock
21	1	M	West Midlands	Medium sheep flock
22	2	1M 1F	South West England	Small beef herd, medium sheep flock
23	1	M	West Midlands	Medium beef herd, medium sheep flock
24	1	F	South West England	Small beef herd, medium sheep flock
25	1	M	South West England	Small beef herd, medium sheep flock
26	1	M	North East England	Medium beef herd, medium sheep flock
27	1	M	North West England	Large beef herd
28	1	M	South West England	Medium beef herd
29	1	F	Wales	Small sheep flock
30	1	M	Wales	Small beef herd, medium sheep flock
31	1	M	Wales	Small beef herd, small sheep flock
32	2	1M 1F	Wales	Medium beef herd, large sheep flock
33	1	M	West Midlands	Medium beef herd
34	1	M	West Midlands	Medium sheep flock

a *Small beef herd <100, medium beef herd 100–300, large beef herd >300; Small sheep flock <400, medium sheep flock 400–800, large sheep flock >800*.

Farmers who took part in the quantitative study and agreed to be interviewed were contacted by phone to arrange a suitable time for interview. All interviews were conducted on the respondents' farms and fieldwork took place between July 2018 and December 2018. The interviews were conducted by CD, lasted between 30 and 45 min, were digitally recorded and then transcribed verbatim.

The semi-structured interview guide was informed by the literature on risk and decision making (35) and target setting as a means of influencing behavior change (18). The aim of the interview schedule was to consider farmer risk taking regarding antibiotic use and resistance using the sociological domains presented by Zinn (35). Zinn reviewed the existing body of risk research to systemise the sociological domains of risk taking behavior into control, motives, reflexivity, and identity. Risk taking can depend on the level of *control* a person has or perceives. They make take risks in order to regain control of a situation, or to confirm their level of control over a situation. Level of control is often entangled with trust where social relationships are involved. Peoples risk taking can be driven by their social *motives* such as feeling of excitement, feelings of self-worth or in response to vunerability. *Reflexivity* refers to embedded human belief structures rooted in the social world including habitual risk-taking, routinized risk-taking or normalized risk-taking. Finally, a persons social *identity* can be key to explaining their risk-taking. People may take risks in order to develop their identity, their identity may shaped what risks are deemed acceptable to take or they may take risks to protect their identity. Not all of these domains may be relevant to risk taking regarding farmers antibiotic use or resistance. Instead, we can aim to identify which of these domains are relevant through asking broad questions relating to antibiotic use and resistance. The topics covered in the interviews included general farm practices, antibiotic use, perceptions of current antibiotic use, national reduction targets, perceptions of the risk of antibiotic resistance, antibiotic use on other farms, and perceived responsibility of antibiotic use monitoring. During the discussion of targets for antibiotic use, a show card was used to indicate the national 10% reduction target set by the RUMA Targets Task Force for the sheep and beef sectors (18).

The transcribed data was coded using the constant comparative method (36) to identify emerging categories of data. Analysis was supported by the use of NVivo (NVivo qualitative data analysis Software; QSR International Pty Ltd. Version 12, 2018). Validity and reliability was established through rigorous record keeping, reporting of data collection, analysis and then verification of findings by two researchers with transcripts being read and re-read by two researchers CD and AR. The researchers' analyzed the data in matrices for each respondent until data saturation was achieved. There were three steps to the coding process: initial coding, focused coding and theoretical coding. Initial coding was the first step where many codes are produced. Focused coding then narrowed down the number of codes by selecting the most suitable ones. Finally, connections are made between codes to produce categories. Categories can then be related to each other to establish a script. Enticott and Vanclay (37) describe scripts as a unique sequence of actions that define a well-known situation—scripts are learned through people's perceptions of the regular and repeated features of the world. Sociological scripts stress the flexibility and capacity of the script writer to learn and update them thus in making sense of a particular situation people develop appropriate roles for themselves that match the given situation to their actions (37).

The scripts were then used to assess the RUMA targets set for the sheep and beef sectors. This was based on recommendations from a policy analysis of target setting in the NHS (38). The policy analysis introduced five tests that must be met for targets to be appropriate and effective. If these tests are met then target-setters are more likely to achieve the desired outcome. The scripts were evaluated against these five tests to understand how RUMA targets can be more effective in the future and consequently aid behavior change around antibiotic use.

The study was approved by the University of Nottingham School of Veterinary Medicine and Science Ethics Committee (no. 1850 160916).

## Findings

Scripts have been described as moral resources used by farmers to account for and justify their management of disease and as a means of farmers re-constructing their identity (39). They are situationally contingent and socially constructed (37) and have been described as a culturally shared expression, story or common line of argument which provides an explanation for a particular course of action. Respondents articulated four main scripts or lines of argument when discussing their management of the risk of antibiotic resistance on their farms:

- Antibiotic resistance—the script of an emerging threat
- Good farmer habitus—the script of experienced, knowledgeable farmers
- Adopting preventative measures—the script of controlling risk and adopting the precautionary principle
- Taking responsibility for risk—the script of risk, defense, and othering

### Antibiotic Resistance—The Script of an Emerging Threat

When asked about their current use of antibiotics respondents reported administering antibiotics for a wide range of problems. In sheep these included mastitis, lameness, watery mouth, abortion, joint ill and navel ill. In beef, antibiotics were mainly used for respiratory disease, lameness, and eye infections.

Given the range of infections that were being treated with antibiotics respondents were asked about the potential of developing antibiotic resistance on their farms. When addressing this question they amplified the potential risks associated with antibiotic resistance identifying factors that had the potential to risk not only their reputation as a good, profitable farmer but also to the health and wellbeing of their animals:

“*Well, it could end up catastrophic! I suppose because if you can't treat something that spreads and gets out of hand, I suppose you move onto the antibiotic so you're moving up the ladder all the time onto more expensive drugs, yeah I mean… it'll be bad for both animal and for pain wise, it'd bad for the farmer financially, won't it?*” Respondent 26 (Sheep and beef farmer)

“*Well, they could be very serious if you have a pneumonia outbreak that you can't treat then- what are you going to do? You could lose 30 animals in the blink of an eye. Really so it's devastating potentially if we suddenly found we had no antibiotics that we could use*.” Respondent 6 (Sheep and beef farmer)

“*Obviously, antibiotic resistance would have a massive impact then on the long-term profitability of farm animals. It is something that we have to be very, very careful—through overuse or through incorrect administration, under dosage we could contribute to antibiotic resistance. Weigh your animal before administering so that you are administering the correct dosage*.” Respondent 11 (Sheep and beef farmer)

Antibiotic resistance was perceived as a potentially catastrophic risk that would need ongoing risk assessment and management to protect the animals and their livelihoods.

Twelve respondents also expressed fears that they may already have observed signs of resistance on their farms and over half felt they were at high risk of developing resistance given they had encountered difficulties in treating infectious diseases in their animals:

“*When we used to have bottles they'd last for… they'd go out of date before we use ‘em, now it’s sort of when you start to use ‘em you use ‘em and 20% of times you use ‘em they just don't have effect now we're starting to see; the antibiotics haven't got the fight power against the disease what they used to have*.” Respondent 2 (Sheep and beef farmer)

“*I bought a calf several years ago now which had resistance to Marbocyl, ‘cause it had scours, resistant to Marbocyl, and caused quite a few problems until we found out what the problem was and obviously got on top of it and it's been fine since, but yeah, I don't want repeats of those situations*.” Respondent 17 (Sheep and Beef farmer).

Their scripts suggested that the Specter of antibiotic resistance was challenging their sense of identity as a good farmer undermining their confidence in their ability to identify and treat infections with certainty and challenging their ontological security i.e., their sense of continuity and order in life (40, 41). They reported that they were sometimes unable to determine whether they had wrongly identified disease or were subject to antibiotic resistance:

“*I did think when I bought the bunch of sheep that introduced the CODD onto the farm, I was thinking we were getting resistance to Oxytetrin or Terramycin, whatever you wanna call the drug, because the sheep's feet… when we were treating lame sheep they weren't responding to the treatment but it wasn't that actually, it was the fact that we were not treating footrot anymore and we were using a drug that didn't really control CODD. Anyway, but it just took me a little while to cotton on to the fact that I was treating a different problem. So we tend to use Zactran now which is very expensive but very effective*.” Respondent 6 (Sheep and beef farmer).

“*And they haven't cleared up, but I don't know if that's resistance or not or just I'm probably assuming it's one type of lameness and it's another type that that antibiotic isn't, yeah. So no, I don't know if we've got any resistance to be honest*.” Respondent 21 (Sheep farmer)

Respondents were concerned that rising antibiotic resistance due to inappropriate use in the industry may cause restrictions on what and how antibiotics were used on farms. This posed a threat to the farmer's way of life and the welfare of their animals. Farmers felt that it could get to the stage where only veterinarians could administer drugs to the animals on their farm. This could cause a breakdown in the trust between farmers and veterinarians, or not allow trust to develop to begin with.

“*Even if legislation came in that we had to have a vet to administer antibiotic use that would definitely cause a huge maybe reduction in the use of antibiotics. Economically it's not possible to profitable in keeping sheep and having your vet out to treat or administer antibiotics for every six sheep, so I would have thought then that there would be massive welfare issues, especially within the sheep sector if antibiotic usage was restricted to veterinary surgeons only*.” Respondent 11 (Sheep and beef farmer)

“*But it's going to get very awkward when it's lambing time and you need Pen & Strep or something for a ewe with a difficult lambing and you've got to go all the way down to (town) and get it in a syringe and bring it all the way back again. That's the death of farming. They've got to trust us*.” Respondent 15 (Sheep and beef farmer)

Farmers were also concerned that rising antibiotic resistance within the sheep and beef sectors could damage the public perception of agriculture and consequently they could face blame for antibiotic resistance in humans.

“*It's also the public perception of agriculture as a whole; if we're seen as a hotbed for developing resistance to antibiotics how long is it before that transfers to a human population?*” Respondent 31 (Sheep and beef farmer)

Overall, respondents presented a story of a major risk or threat to the health of their animals which was changing their relationship with risk, undermining their confidence in their ability to manage the risks encountered and threatening their ontological security—that is their sense of continuity and order in life and in the consistency of the surrounding social and material environment (41).

### Good Farmer Habitus—The Script of Experienced, Knowledgeable Farmers

Habitus is formed in the context of people's social locations and inculcates them into a world view which is based on and reconciled to their position, thus serving to reproduce existing social structures (31, 33). Habitus is developed in dialogue within context and on the rules of the game, which is certain rules they have the play by to gain cultural, social, and economic capital and is subject to change over time (33).

Most respondents reported that they felt that they had the skills to identify infections and administer antibiotics or common conditions that they encountered, such as pneumonia or footrot. They argued that they were experienced stockpersons and capable of assessing symptoms, identifying the disease, and deciding on a course of appropriate action. They reported that their assessments drew on their intuition, situated knowledge and experience to assess risk to the health of the animal:

“*Well it's just years of experience of doing it really, you just know when an animal's ill and it needs antibiotic treatment….It's either severely lame or got an infection from a wound or has got breathing difficulties…So an animal that stands out to us absolutely as having pneumonia we put in the crush, you can see its breathing, you can even listen to it if you wanted to. If you're sure you know what you've got, we would treat that ourselves…Take its temperature, usually if it's pneumonia it'll have a high temperature which will give you an indication of infection there, so yeah you know what it is*.” Respondent 1 (Beef farmer)

“*Well, if you've been around animals for long enough and if you've been brought up on a farm most farmers can identify a sick animal at a first glance. Yeah. You only need to look at their ears and eyes really, and the way they walk*.” Respondent 11 (Sheep and beef farmer)

They suggested that most of the time their knowledge and experience was sufficient for them to decide if antibiotics were needed and that their confidence levels in terms of using antibiotics were high:

“*I feel very confident that I know when (to give antibiotics) 90% of the time I'll know when to use them and the 10% of the time I am unsure I have full confidence in my vet*.” Respondent 11 (Sheep and beef farmer)

“*Oh, very confident because I know what a lame sheep looks like*.” Respondent 25 (Sheep and beef farmer)

The treatment of common animal illnesses such as lameness and pneumonia were constructed as an ordinary event and part of the everyday activities on farms. The identification and treatment of such common diseases was learned through their upbringing on farms and embedded in the cultural knowledge of farming:

“*Antibiotics, just footrot in sheep, this time of year it's one of my biggest tasks probably doing sheep work; every time you get a bunch in there's always a sheep lame*.” Respondent 17 (Sheep and beef farmer)

“***P2:*** *You're always going to get lameness*.

***P1:*** *You can identify the lameness so I suppose you'd have to say lameness. You get other things crop up from year to year but lameness will always be… or is always just an underlying one*.” Respondent 22 (Sheep farmer(s))

Farmers considered they were experts in identifying and treating common diseases. They felt that treatment practices had changed very little over time and that it was very rare they would encounter a disease that was unknown to them. Hence, they seldom called their veterinarian out to visit the farm.

“*There's no new real need for them to come out to sheep… You know, if you have a lame sheep you know what it is and we've had vets in the past tell us what to do so it doesn't really change*.” Respondent 10 (Sheep and beef farmer)

“*Not in general healthcare I wouldn't say, maybe on something new because obviously there's the price of the vet and he's only going to probably say the same thing as what we would do, but if it's something new and we didn't know, yes the vet should be involved to understand it*.” Respondent 9 (Sheep and beef farmer)

Farmers believed that their veterinarians trusted their capabilities to identify and treat their animals. For example, one farmer expressed that their experience as a farmer meant that their level of knowledge on their animals' health was on par with less experienced veterinarians.

“*I had a cow last year in the cubicles, she was unwell, she got quite irritable and I rang up the vets and said, ‘Look, I don't know what this is.’ The head vet came out, the senior vet because he said, ‘You know, if you don't know what's going on then there's no point in sending one of the junior vets*.”’ Respondent 13 (Sheep and beef farmer)

Respondents painted a picture of themselves as self-assured, competent, and highly experienced farmers who were capable of managing disease risks appropriately. They were able to draw on a range of internal resources to support their decisions. Such resources were part of a collective of skills and expertise acquired as part of their habitus.

### Preventative Measures—The Script of Controlling Risk and Adopting the Precautionary Principle

Shortall et al. (34) suggest that farmers will strive to be “good farmers” according to the rules of the game—which in the case of reducing antibiotic resistance would be minimizing the use of antibiotics. Early literature on good farming indicated that farmers could be resistant to changing their ideas and ways of working (33). Their habitus and their cultural capital (in this case ability to identify infection and administer antibiotics) ensured that their behavior remained consistent and unchanged. However, when facing new risks that potentially threaten their animals, their livelihood or ontological security (as a good farmer) farmers will change their attitudes and re-negotiate perceived good farming standards (34).

It was evident that respondents feared developing antibiotic resistance believing it would have an extremely detrimental effect on their businesses and their animals. For farmers their farm work represented a central aspect of their life. Any changes to their routine practices needed to be considered carefully:

“*It isn't a case of them feeling that by doing something might risk their business and in farming risking the business is also risking their life, so the consequences are far greater in farming*.” Respondent 24 (Sheep and beef farmer)

The prospect of antibiotic resistance was considered risky enough for farmers to start adopting new practices to minimize this risk. They reported using alternative practices in order to protect their animals and reduce the need for antibiotics. They had begun to think about or use alternative strategies for managing disease on their farms. Strategies they reported included:

**Improving biosecurity**

“*Well, the calves—sourcing from clean herds that give good colostrum and good healthy calves and do not mix them with any other animals from any other farms and have them in separate sheds, separate places*” Respondent 2 (Sheep and beef farmer)

**Managing animal health**

Farmers reported managing animal health by using alternatives to antibiotics including vaccines to prevent disease, anti-inflammatory drugs to aid with pain relief and culling to avoid breeding genetically prone animals and stop disease spread:

“*Vaccines as much as you can. That's our theory. I know people say they cost a lot of money but just so much pleasanter if they're not ill, isn't it? No, we've just got into the habit of vaccination*.” Respondent 26 (Sheep and beef farmer)

“*Sheep wise I think you have got to cull the worst ones, possibly the carriers of any disease, footrot and things like that ….I don't know I can't really.Yeah, there's not much else you can do is there?”* Respondent 30 (Sheep and beef farmer)

“*Cause I use anti-inflammatories as well so I try and cut down the use of antibiotics by using anti-inflammatories to help and hit ‘em hard in one go rather than continuous use of antibiotics. I try not to keep injecting with antibiotics*.” Respondent 3 (Sheep and beef farmer)

In some cases it appeared that efforts to keep antibiotic use to a minimum had a negative impact on animal welfare. This was particularly the case for the treatment of lameness in sheep. Although prompt treatment of all sheep with footrot with injectable antibiotics is recommended by industry (42), some farmers were only treating severely lame sheep with injectable antibiotics.

“*We only use antibiotics for the ones with maggots in ‘em and the hoof falling off*.” Respondent 29 (Sheep farmer)

“*Footrot is the major bugbear but like I said, we inject if they're really, really bad, if not you just trim and spray it and put ‘em through a footrot bath. Yeah, we're not ruthless with it*.” Respondent 30 (Sheep and beef farmer)

Respondents presented a picture of themselves as farmers who were actively engaging in preventative measures, who were signed up to the “precautionary principle” as a way of managing risks associated with infections amongst their animals. Nevertheless, the strategies mentioned did not appear to be part of a coherent plan to manage risk but rather a risk reduction menu from which they could choose a risk reduction option. In adopting these strategies they sought to maintain their sense of ontological security, their self-identity as a good farmer.

### Taking Responsibility for Risk—The Script of Risk, Defense, and Othering

Targets are a set of directed principles to identify the individual steps necessary to achieve a common goal. Given the level of concern about antibiotic resistance expressed by the respondents targets could potentially provide a benchmark for action, foster accountability and support them to achieve the goal of reducing antibiotic resistance on their farms. A target to reduce antibiotic use by 10% was set for the sheep and beef sectors. However, when respondents were asked if they considered the specific reduction aims within the RUMA targets to be feasible for sheep and/or beef farmers—they identified a range of challenges that they might face in attempting to achieve the targets. Though the RUMA targets are for sector-wide antibiotic use and are aimed at a national level, most respondents reflected on this through their own individual farm use.

The notion of having to reduce their antibiotic use by 10% was not an idea that resonated with respondents as they believed that their usage was already lower than the target set. They provided a number of justifications for challenging the idea that they needed to reduce their use and for deflecting responsibility to others.

They defended their position as very low users of antibiotics who had already made such changes and argued that they would not be able to successfully reduce them any further.

“*Reduce mine—I don't think I could reduce it much more than I'm doing because I would say I'm probably using a very, very small percentage to most sheep farmers already because we've stopped using it. Yeah, we've already stopped using it in that sense. I mean I'm sure we could reduce it even more by probably making it even cleaner in the lambing sheds. So reducing what we're using I'm sure I can reduce it more going forward but to reduce it by 10% is probably a bit extreme because I'm not actually using that much anyway now because we changed our practice probably five years ago already. But obviously we'll carry on and try and reduce it, reduce it, reduce it but 10% is quite a lot when you're only probably using four bottles a year*.” Respondent 34 (Sheep farmer)

“*Well, 10% of a lot would be achievable but when you don't use a lot I think it becomes more difficult. So we'll have to wait and see on that one. I think it could give us problems in that we're starting at a low point anyway, so as I say, we'll have to wait and see*”. Respondent 13 (Sheep and beef farmer)

Antibiotics were reported as being used as a last resort or only in emergency situations so any attempt to further reduce their use further would put their animals' health and welfare at risk:

“*I couldn't really reduce it at all without losing stock- I only really use antibiotic when an animal is ill so it would result in loss of animals*.” Respondent 4 (Sheep and beef farmer)

*Well, we only use them when they're necessary so you're gonna have problems with disease spreading and having a worse problem*.” Respondent 8 (Beef farmer)

“*If you get a major breakdown of, say for instance pneumonia, you've gotta treat and it's out of your control and you've just gotta run with it because the welfare issue is the priority rather than the reduction. It's nice to have a reduction but the overwhelming animal welfare is priority to that*.” Respondent 28 (Beef farmer)

The alternative of risking further or ongoing disease amongst their animals was not a risk that they could justify. Nor was a potential risk to their reputation that could result from allowing sick animals to remain untreated. Such action was considered irresponsible in terms of animal health. Their responses appeared to be based on an emotional attachment to their animals and a fear that they could be seen as irresponsible or bad farmers:

“*I think it would be quite bad practice not to use what I use now because it would look like I wasn't caring at all. So somehow there's a line between using it irresponsibly and using it because you need to*.” Respondent 34 (Sheep farmer)

“*It doesn't look very good ‘cause we've got a lot of footpaths and there's always somebody looking over the fence so you've gotta be careful what you do and it's in the best interests of the animal to be healthy and walking around on four legs instead of three*.” Respondent 2(Sheep and beef farmer)

“*I do worry that people looking from the outside say, “Actually, he's got lame sheep there, he's not treating them“*”. Respondent 17 (Sheep and Beef farmer)

Respondents reported additional challenges that they felt would prevent them from protecting their livestock. They suggested that they did not necessarily know the risks they were taking when “buying in” animals that were sick or carrying resistant organisms and this made it more difficult for them to optimize or reduce their antibiotic use for newly purchased animals due to a lack of information about their disease and treatment status:

“*Livestock is moving between farms all the time so you don't know what you are buying in when you buy breeding stock, so yes, I mean it is a concern yes*.” Respondent 6 (Sheep and beef farmer)

“*I wouldn't know that, would I, ‘cause you don't get a history of what animals are treated with when they come*.” Respondent 19 (Beef farmer)

“*I guess the problem we have is that we no control on what happens before they come on farm, I think a lot problems, particularly with pneumonia I think are historic.so if they happen to have pneumonia when they're younger they're more susceptible to get it later on in life, aren't they? …There's no point saying 10% less when someone's probably using 20% more than us, that'd make a 30% difference*.” Respondent 8 (Beef farmer)

Importantly, trying to interpret and measure progress against the targets, particularly without knowing their baseline usage was considered outside their remit or skill base so they would not be able to meaningfully reduce the risk associated with over use of antibiotics:

“*So as a farmer I don't know what 10 milligram per PCU means*.” Respondent 14 (Sheep and beef farmer)

“*I don't think everyone knows what we are using now to be able to reduce it by 10%. I don't know how you're gonna use the target you've got at the moment ‘cause we haven't really got a baseline target at the moment*.” Respondent 5 (Beef farmer)

“*Yeah, depends on what your usage level currently is, if it's really low then it's harder to get it down by 10%, isn't it? We're always trying to reduce it for sure, because it reduces costs and sick animals don't perform. So, if you can prevent ‘em from being sick in the first place it's a win-win situation. Yeah, I'd have to… we probably can reduce it, whether we can do it by 10%—if we were a big user, you know, there's a lot of farms where it's easily achievable*.” Respondent 32 (Sheep and beef farmer)

Having to interpret statistics so they could reduce their antibiotic use by 10% would act as a barrier to change and potentially be at odds of their habitus and their concept of themselves as good farmers who prioritize the well-being of their animals and the viability of their farm. A lack of evidence or information about how the targets were being measured prevented them from being able to decide if they were contributing to achievement of the national target to reduce usage. They reported feeling unable to fully understand the nature of targets and how to turn them into action. In such circumstances, it was difficult to “own” the targets and they sought to locate responsibility for achieving them with others.

#### Othering

Given the challenges respondents reported in attempting to reduce antibiotic use they sought to shift responsibility for the achieving the targets and blame for not achieving them on to others. Othering involves defining or defending self-identity by distancing oneself from individuals or other groups who are excluded or regarded as posing risks to self-identity (40). Individuals use social skills and their judgements of situations and especially of others, which might be based on hearsay or intuition and shaped by shared experience or habitus (32). Perceptions of risk among people sharing the same cultural context are related to the groups' legitimizing moral principles—thus “others” are often identified as threatening the mainstream (43).

Farms that overused antibiotics were framed as poorly run and held responsible for increasing the risk of antibiotic resistance:

“*Poor management. Simple as. Not knowing what they're doing properly. Probably not seeking professional advice through their vets. So, using excess, did you say, of antibiotic? Well, if they've got a major problem then perhaps on certain years they've got to, I mean who's to say I might have watery mouth next year. But, generally that seems a bit like poor management, poor husbandry and not consulting the vet enough. I don't know*.” Respondent 21 (Sheep farmer)

“*Too high a stocking densities, mixing of age groups within buildings, unvaccinated animals coming, where they haven't got a clue where they've come from, poor hygiene and general standards so cattle aren't looked after well… full of rats and other things and various things that can spread disease and issues. Blanket antibiotic treatments and stuff like that*.” Respondent 1 (Beef farmer)

“*Well the ones that do blanket treatment for abortion first of all. There are others that routinely use Spectam in every new-born lamb and penicillin when it could be achieved through better nutrition for the ewe. Well, if you have problems with mastitis that could be bred out, that is… you've not to chase yields as much and to not breed from ones who've had mastitis in the past, that could be reduced in the dairy industry. Yes, there are people using too much, definitely. And there are some who under… don't give the prescribed dose, give a half dose and I'm sure that increases resistance, does it?*” Respondent 10 (Sheep and beef farmer)

In describing poorly run farms as problematic, respondents were able to distance themselves from this type of risky behavior, even though some of the problems they attributed to others had also been described in their own experiences of the difficulties they faced in achieving the targets. Other farmers were identified as the risky others—who posed a threat to farming. Poorly run farms became unsafe places which threatened animal health and farmers' livelihood.

Responsibility for reducing the risk associated with antibiotic resistance was also ascribed to veterinarians. In the UK all antibiotics for veterinary use must be prescribed by a veterinarian. The Royal College of Veterinary Surgeons (RCVS) Code of Professional Conduct states that “The animal or herd must have been seen immediately before prescription or recently enough or often enough for the veterinary surgeon to have personal knowledge of the condition of the animal or current health status of the herd or flock to make a diagnosis and prescribe” (44). Whilst, all respondents stated that they obtained their prescriptions and antibiotics from their veterinarians, they indicated that their contact with the veterinarian could be sporadic and may not necessarily occur at the time of illness in the animal:

“*The vet is not involved generally because if you get an animal sick when I go round them in the morning I'm not going call the vet before I do it (administer antibiotics). If we've got a really sick animal that the vet has come out to then that becomes the vet's choice but it is very unusual that we'd have a vet out to an animal*.” Respondent 34 (Sheep farmer)

“*But sheep farmers don't keep running down the vets, you know, we can sort out our own lambing, we hardly ever go down for a lambing. The vet comes out for a TB test or maybe a calving but they're always too busy to talk to you*.” Respondent 15 (Sheep and beef farmer)

Although they reported having little contact with their veterinarians, when identifying and treating infections respondents suggested that veterinarians should shoulder responsibility for helping farmers to achieve antibiotic reduction targets:

“*No, the vets are the only ones that are gonna tell you that. And hopefully by doing this you're gonna tell me whether I am or not (over using) … the vets gonna monitor it anyhow ‘cause you've gotta buy the antibiotics from the vet, you can't get it any other way*.” Respondent 2 (Sheep and beef farmer)

“*Well, I would've thought the vet would be the best in the know, won't they? They provide us with the antibiotics so they're in a good position, aren't they, to monitor antibiotic use*.” Respondent 19 (Beef farmer)

In attributing this responsibility to their veterinarians, respondents appeared to be deflecting responsibility away from themselves alone suggesting that vets should play a significant role in the process. They believed that veterinarians were more knowledgeable about antibiotic use and in control of the situation because antibiotics must be prescribed by them. However, some farmers noted that antibiotics could be too easily accessible from the veterinarian, and some were confused about the conflicting information received from veterinarians.

“*It's too easy to go to the vet and ask for something and they just give it to you and can be used pretty easily*.” Respondent 17 (Sheep and beef farmer)

“*So I know that there are vets that perhaps maybe that give out the antibiotics without good reasoning and without a good understanding, so*.” Respondent 1 (Beef farmer)

“*Sometimes we're slightly conflicting ‘cause we wouldn't have always vaccinated lameness, we'd have trimmed and put them in a footrot bath, unless they were really bad and then we would've, but now we're told we've got to use antibiotics. So it's sort of slightly conflicting information coming out, isn't it?*” Respondent 21 (Sheep farmer)

Respondents presented a story of themselves as good farmers who already met the targets set and who would be putting their animals and their reputation at risk if they tried to reduce them further. They portrayed themselves as responsible actors and distanced themselves from accountability for reducing antibiotic use by shifting blame onto poorly run farms and responsibility on to veterinarians. Thus, their responses to risk sought to maintain symbolic boundaries with the farming community particularly in relation to self and others (40).

## Discussion

This is the first study to the author's knowledge to provide insights into the way in which sheep and beef farmers in England and Wales view the feasibility of antibiotic reduction and the risks posed by antibiotic resistance. The present study used a qualitative approach and aimed to build on the relationship between risk and habitus to gain an understanding of sheep and beef farmers' decisions and actions relating to reducing antibiotic usage. Through gaining this understanding, the study aimed to support successful behavior change, policy implementation, and the reduction in the risk of antibiotic resistance. The aim was not to quantify opinions and the results do not suggest that every opinion reported is held by every sheep and beef farmer in England and Wales. However, using this approach we were able to capture the perceptions, beliefs and motivations that underpin behaviors regarding antibiotic use and the potential for its reduction.

Lupton (45) argues that both emotion and risk are inevitably configured by social and cultural processes and through interaction with others, material objects, space and place. Emotion and risk assessments are fluid, shared and collective underpinned by trust and intuition. She suggests people use an “Emotion—Risk Assemblage,” which is a combination of ideas and concepts brought together to assess and manage risk or uncertainty (27, 45). In doing so Zinn (27) suggests that risk management strategies devised using a cultural perspective cannot necessarily be identified as either “rational” or “irrational.” Thus, when individuals weigh up risks they are deciding how risk phenomenon cohere with their values about what is acceptable and what is threatening. They utilize “in between” strategies such as trust, intuition and emotion to manage them (26, 27). The interpretations of risk presented by Lupton (45) and Zinn (27) can be used to understand the farmers views of risk around antibiotic reduction and antibiotic resistance. Farmers in this study drew on their emotional ties to their animals, their habitus, and their sense of ontological security—as good farmers—to defend their practices and to blame others for the problem. Their views of problem farms were underpinned by emotions of fear and blame and they were seen as dangerous places (40, 43). They did not follow rational risk assessment and management strategies to deal with the potential risk of antibiotic resistance but were aware of and concerned about the potential threat to their animals and livelihood posed by antibiotic resistance. Respondents adopted a broad precautionary approach and engaged in risk management strategies associated with biosecurity. However, they did not understand the scientific basis of targets associated with reducing antibiotic use on their farms nor did they feel confident to calculate how much of a reduction in antibiotics would be needed to reduce their use. Thus, their scripts revealed that their habitus—as good farmers—influenced the way they sought to justify action and or inaction in relation to reducing antibiotic use on their farms. They described a risk response that was based on an assemblage of beliefs, ideas, emotion, intuition, and logic of practice (32)—a risk-emotion assemblage (45). Their scripts acted as a resource to normalize actions and deal with issues of accountability and reputation management (46).

Policy makers are increasingly acknowledging that the elimination of all risk presents a major challenge. Focusing on systems that more accurately identify and categorize risks and provide programmes for handling and reducing risks are considered more likely to be effective (23). The insights revealed through the scripts in this study have important implications for policy makers who adopt rational approaches to bringing about change. They illustrate how strategies for change based on evidence or on the precautionary principle could be less effective than desired. In particular this study highlights the complexities surrounding the setting of numerical targets for reduction of antibiotic use in the beef and sheep industry. The setting and monitoring of targets is one way in which governments can provide leadership, guidance, and strategic direction to achieve a reduction in risks through behavior change. Targets are expected to motivate people to achieve goals with appropriate milestones, to foster accountability and provide motivation. Nuti et al. (47) suggest that governance based on targets is a form of indirect control which requires selecting the appropriate number of indicators to measure the objectives and choosing a rigorous principle to define which indicators should be considered as priority. Targets are extensively used in UK policy particularly in relation to improving health and well-being and increasing the efficiency of hospitals. Although targets have met with success evidence suggests that this approach can also be accompanied by unintended negative consequences. For example, in the UK the 4 h waiting target for people attending Accident and Emergency services, whilst generally successful has resulted in poorer care for some patients (48). Thus, although targets can change people's behavior in order to the meet the target they may not choose to do this in the way the target setter intended (49). In addition, Elkan and Robinson (50) argued that targets focus action on those things that are most easily measured and can foster complacency on the part of providers who have already achieved target levels of performance and defensiveness from those performing badly.

Targets are just one means to achieve progress against a priority, but not all priorities lend themselves to a target. Before deciding on a new national target or whether to have a target at all, policymakers need to consider whether it is the most effective and appropriate means of achieving the desired outcome (38). Berry et al. (38) identified key questions that would need to be addressed to assess the suitability of targets and the data presented in this study provides valuable insights into the potential challenges faced in reducing antibiotic use on sheep and beef farms in the UK. These are discussed below:

**Firstly, is there a widely RECOGNIZED and pressing problem, which requires policy action at a national rather than just local level?**

Globally and nationally there is a persuasive need to address the unnecessary use of antibiotics to ensure responsible use in humans and animals (both production and companion). The UK sheep and beef sectors are traditionally low users of antibiotics but, nevertheless, have some “hotspots” requiring action including lameness, abortion, and neonatal diseases in sheep and pneumonia in beef cattle. Additionally, the usage figures taken from convenience samples and reported in the latest VMD and RUMA reports suggest that usage in parts of the beef sector may be higher than the dairy sector (11).

It was evident through respondents scripts on *antibiotic resistance as an emerging threat* that the sheep and beef farmers interviewed in this study were aware of the risks associated with antibiotic resistance stating that it could be catastrophic both for their livelihoods and reputations if they were to develop resistance on their farms. Some feared they may have already observed antibiotic resistance, and most felt at high risk of experiencing it in the future.

**Secondly, is the problem likely to be amenable to action by those who are accountable for the target?**

Farmers in their scripts of *experienced, knowledgeable farmers and of risk, defense, and othering*, provided a strong rationale for not cutting back on antibiotic usage. They believed that they were very low users already—only using them in life and death situations—and that that a reduction in use would increase the mortality in their flocks or herds and in turn, potentially risk their reputation as a good farmer. Given that they reported that they were not able to determine their exact use or calculate a 10% reduction their beliefs were based on subjective views.

Thus, while it was evident that the problem of antibiotic resistance may be amenable to action by the respondents their strong beliefs about the individual animal being a priority and their concerns about being categorized as a bad farmer may result in action that will limited the potential to reduce antibiotic resistance. Nevertheless, their scripts on *controlling risk and adopting a precautionary principle* suggested that they were signed up to the “precautionary principle” and that they had adopted alternative measures to reduce the risk of infection on their farms.

However, they did not appear to sign up to the idea that they could be held accountable for the overuse of antibiotics in farming or adopt the target of reducing their antibiotic use by 10%. Their risk rationales were fluid, relational, and contextual (45). The farmers' cultures were located within specific spaces (their farm) and Lupton (45) suggests that features of space and place are important in the production and expression of emotional states. Farmers expressed concerns that if they tried to reduce their antibiotic use any further they would put their animals and reputation at risk. These fears were based on emotional ties to their animals on the one hand and their identity as a good farmer on the others.

Experts' attempts to change risk taking behavior often fail as they do not engage with peoples' identities, the social rooting of risk taking and the social power structure. Respondents suggested that farmers who had more intensive systems, or had poorly run farms with high stocking rates were most likely to be the ones who were creating the risk associated with antibiotic resistance. The act of other-blaming around the responsibility of antibiotic resistance by both livestock farmers and veterinarians has recently been highlighted by Golding et al. (51), where blame was also directed at other farmers with poor antibiotic practices. Notions of self and risky others can be underpinned by the emotions of fear, distrust, hate, blame but rather than being irrational Zinn (20) suggests that they are simply a different intelligence about the world.

**Thirdly, do the necessary resources to take action already exist or can they be developed?**

The RUMA targets for increasing uptake of vaccinations for footrot and abortion have not been maintained at the proposed rate (11). Farmers in this study did not feel they had all the necessary resources to take the action needed to meet the antibiotics reduction targets. In their script on *taking responsibility for the risk* they explained that when they bought in new animals they were not supplied with their health and vaccination history making it difficult to decide on the action to take if the animal became ill. Availability of medicine records could be improved to make it easier for farmers to inspect the medicine history of potential animal purchases and this should improve as plans develop for the UK centralized medicine hub. A livestock information programme is also being developed through an industry-government partnership in order to improve animal traceability (52). Electronic medicine books are already available for pig producers with various groups exploring the development of equivalent tools in the cattle and sheep sectors (11, 53).

The main source of information to support antibiotic reduction for farmers in this study was the veterinarian. However, respondents suggested that they had limited contact for diagnosing and treating infectious disease, as also indicated in previous studies (54, 55). This implies antibiotic reduction may reach an impasse if veterinary visits do not become more regular on farms. At the moment, lack of contact with the veterinarian, or infrequent veterinarian visits, is considered normal or acceptable in the sheep and beef sector. Infrequent veterinarian visits may be even seen as a symbol of a good farmer as veterinarians are only used in emergencies or with the emergence of new diseases. Whilst quality assurance schemes in the UK require an annual veterinary visit to the farm with a herd or flock health review (56), the social conditions around the normal frequency of veterinarian visits needs to change for targets to be effective. Some respondents also felt that antibiotics were too easily accessible from the veterinarians and antibiotics could be prescribed without thorough reasoning. This highlights a potential lack of reflexivity around the use of antibiotics in the sector. If veterinarians do not attempt to question farmers who ask for antibiotics, then the farmers themselves might not reflect on and examine their own antibiotic use.

Nevertheless, within their script of *risk, defense, and othering* they suggested responsibility for achieving a reduction in antibiotic use lay with the veterinarian so there is potential for veterinarians to take a lead in supporting farmers. There is an argument for the increased use of proactive veterinary-led flock or herd health planning that encourages the application of appropriate preventative measures to manage disease risk and use antibiotics responsibly.

**Fourthly, can changes in performance can be adequately measured?**

RUMA (57) reported that data collection to measure progress remains challenging. Farmers in this study reported not understanding the measurements and were confused about how a reduction of 10% could be measured and achieved. Most respondents believed that the veterinarian should take responsibility for monitoring antibiotic use because they were the ones supplying the medicines. However, at the same time respondents stated that they had minimal contact with their veterinarians for the treatment of their sheep and cattle. Farmers also indicated that they did not have the skills to accurately measure their progress against the numerical targets. To ensure performance is adequately measured it is important that farmers and veterinarians work in partnership, with the farmers supplying farm management data and the veterinarians facilitating the analysis of on-farm antibiotic usage. Zinn (35) suggests that if awareness is lacking or knowledge is inaccurate, education and information strategies might be important for achieving behavior change.

**Fifthly, do the targets align well with what already exists or is planned elsewhere in the system, with minimal negative consequences?**

As the farmers in this study believed that they were using as little antibiotics as possible already, further reductions in antibiotics were seen as being be detrimental to production and animal health and welfare and therefore associated with negative consequences. For example, lameness is an endemic disease in British sheep flocks and the Farm Animal Welfare Committee set targets to reduce sheep lameness to less than 2% by 2021 (42). The five-point plan is a national strategy for achieving this target and one of measures for the control of lameness is to give an antibiotic injection within 3 days of the sheep becoming lame. Consequently, farmers may feel that they have to choose between achieving the lameness targets and achieving the antibiotic targets. Some farmers were minimizing their antibiotic use by only treating severely lame sheep with injectable antibiotics. This indicates that the antibiotic reduction targets could have a negative impact on animal welfare. Thus, messages need to be clearly delivered to farmers that it is entirely appropriate to treat clinically affected sheep with antibiotics with emphasis on prevention by rapid treatment and improved biosecurity, which in turn will reduce the lameness level and lead to fewer lame sheep and further reductions in antibiotic use in the long term. Veterinarians need to ensure that they take the time to explain and provide farmers with context with how using antibiotics appropriately reduces usage in the long term. As this study shows that farmers do not always their veterinarians for advice on lameness, there is also a need to use industry more widely to ensure messages around appropriate antibiotic use are conveyed.

### Implications for Policy

From the evaluation of the farmers scripts against the five tests for effective targets outlined by Berry et al. (38), a number of weaknesses in the current targets are evident. Through identifying these weaknesses, we can determine how the targets can be more effective in the future and consequently aid behavior change around responsible antibiotic use.

Firstly and perhaps most importantly, the majority of sheep and beef farmers have not had their antibiotic use measured officially. Therefore, they do not know how much antibiotics they are using compared to the targets. A numerical antibiotic use target could be counterproductive if most farmers do not know their numerical use. It is suggested that until there is reliable data collection and robust metrics available there should not be a numerical target set for antibiotic reduction in the sheep and beef sectors. Targets should first focus on comprehensive collection of antibiotic consumption data. Other measures of “responsible” use which are already reported in the RUMA reports could be framed as more important until reliable antibiotic use data is achieved (11).

The lack of availability of antibiotic use data also fosters issues with accountability for the targets. As farmers believed that they were low users of antibiotics, they shifted the responsibility for reducing antibiotics onto “others.” From the results of this study it is suggested that the framing of the current targets for the sector should be shifted from *reducing* antibiotic use to *responsible* antibiotic use. At present, the RUMA targets start with an antibiotic reduction target—such as reducing antibiotic use by 10%. This is then followed by responsible antibiotic use targets—such as increasing vaccine sales to prevent disease (11). The targets should instead prioritize the responsible use targets. This will help all farmers feel accountable for the targets.

By emphasizing responsible antibiotic use instead of reducing antibiotic use, this will also help to ease the conflicts faced with other recommendations. The focus on reducing antibiotic use could be especially detrimental to sheep lameness control targets. If antibiotics are to be used responsibly in the sheep sector in particular, the optimal control strategies for lameness need to be highlighted in the report using evidence based reasons, sources, and consequences.

Finally, the resources available to farmers to support their responsible antibiotic use needs to be developed. As veterinarians are the main source of information for farmers, their means of communication could be developed to provide farmers with better resources. The farmers tended to interpret the sector wide targets in an individualistic manner and placed value on their situational knowledge when treating animals with antibiotics. People prefer communication that is tailor-made to them and their values (58). Thus, veterinarians could share knowledge and understanding about responsible antibiotic use with farmers based on their values (e.g., animal welfare, reputation) through personal stories. Knowing farmers values and beliefs requires a strong relationship between veterinarians and farmers, however this cannot happen when veterinary visits to the farm are infrequent. Therefore, there is a need to normalize frequent vet visits on sheep and beef farms and make infrequent veterinary visits appear less acceptable. Overall, regular veterinarian visits to sheep farmers needs to be embedded in the “good farmer” ideal.

## Conclusion

This paper used qualitative methods to explore beef and sheep farmers' perceptions and their management of the risks associated with potential overuse of antibiotics on farms. In particular, the study used script theory to examine the potential influence of farmers' beliefs and behaviors on the achievement of national targets to reduce antibiotic use on farms. The beliefs and behaviors, reported by respondents, are of utmost importance for policy makers to consider in terms of achieving national targets set by the RUMA Targets Task Force. Respondents reported not having the technical knowledge and skills needed to measure antibiotic use and resistance, they believed their use of antibiotics was already low and they were concerned about the potential effect of further reducing use on their business, their animals and their reputations. They deflected accountability and responsibility for dealing with the problem to veterinarians and poorly managed farms. These insights are valuable for policy makers to enable them to set realistic targets, which have research-informed objectives to support farmers and their veterinarians as they aim to make progress in the achievement of the targets. Additionally, the insights may help to form a basis for providing education and training for farmers to mitigate against the risks of antibiotic resistance developing on their farms. This study demonstrates the value of social science research methods in understanding the factors that influence behavior change in farming and provides valuable insights for policy makers tasked with achieving behavior change.

## Data Availability Statement

The datasets generated for this study will not be made publicly available to ensure confidentiality of respondents. The data supporting the conclusions of this article will be made available by the authors, upon reasonable request, to any qualified researcher.

## Ethics Statement

The studies involving human participants were reviewed and approved by University of Nottingham School of Veterinary Medicine and Science Ethics Committee (no. 1850 160916). The patients/participants provided their written informed consent to participate in this study.

## Author Contributions

CD, AR, and JK contributed to conception and design of the study and the themes generation. CD performed data collection. CD and AR performed analysis of transcripts. AR and CD wrote the first draft of the manuscript. JK wrote sections of the manuscript. FL, CH, and LK contributed to the manuscript revision and interpretation of data. All authors read and approved the submitted version.

## Conflict of Interest

FL is a member on the RUMA Target Task Force as a representative of the sheep industry and LK is an employee of AHDB, which is also a member of RUMA. FL and LK did not participate in design and analysis of the study. The remaining authors declare that the research was conducted in the absence of any commercial or financial relationships that could be construed as a potential conflict of interest.
